# LC-MS/MS Screening, Total Phenolic, Flavonoid and Antioxidant Contents of Crude Extracts from Three Asclepiadaceae Species Growing in Jordan

**DOI:** 10.3390/molecules27030859

**Published:** 2022-01-27

**Authors:** Yousef Al-Dalahmeh, Nezar Al-Bataineh, Sara S. Al-Balawi, Jamil N. Lahham, Idrees F. Al-Momani, Mohammed S. Al-Sheraideh, Abdulraouf S. Mayyas, Sultan T. Abu Orabi, Mahmoud A. Al-Qudah

**Affiliations:** 1 Department of Applied Pharmaceutical Sciences, Faculty of Pharmacy, Isra University, Amman 11622, Jordan; yousef.dalahmeh@iu.edu.jo; 2 College of Pharmacy, Al Ain University of Science and Technology, Abu Dhabi P.O. Box 6414, United Arab Emirates; Nezar.albataineh@aau.ac.ae; 3 Department of Chemistry, Faculty of Science, Yarmouk University, P.O. Box 566, Irbid 21163, Jordan; sarass20008@gmail.com (S.S.A.-B.); imomani@yu.edu.jo (I.F.A.-M.); abuorabi@yu.edu.jo (S.T.A.O.); 4 Department of Biological Sciences, Faculty of Science, Yarmouk University, Irbid 21163, Jordan; jamil@yu.edu.jo; 5 Chemistry Department, College of Science, Imam Abdulrahman Bin Faisal University, P.O. Box 383, Dammam 31113, Saudi Arabia; msalsheraideh@iau.edu.sa; 6 Department of Conservation Science, Queen Rania Faculty of Tourism and Heritage, The Hashemite University, Zarqa 13133, Jordan; A.S.Mayyas@hu.edu.jo; 7 Department of Medical Analysis, Faculty of Science, Tishk International University, Erbil 44001, Iraq

**Keywords:** total phenolic, flavonoid, antioxidant properties, *Calotropis procera*, *Peruglaria tomentosa*, *Pentatropis spiralis*, Aclepiadaceae

## Abstract

This study aimed to evaluate the antioxidant activity and total phenolic content (TPC) and total flavonoid content (TFC) of crude extracts obtained from three *Asclepiadaceae* species, namely, *Calotropis procera* L., *Peruglaria tomentosa* L., and *Pentatropis spiralis* (Forsk.) Decne. Both butanol and aq. methanol extracts of the three species showed the highest amount of phenol and flavonoid contents, which exhibited the greatest antioxidant activity in the scavenging of 2,2-diphenyl-2-picrylhydrazyl free radical (DPPH), 2,2′-azino-bis(3-ethylbenzothiazoline-6-sulfonic acid) diammonium salt radical cation (ABTS), ferrous chelating effect (FIC), and hydroxyl radical (HDR) assays. Phytochemical screening of the extracts revealed the presence of alkaloids, tannins, sponins, flavonoids, terpenoids, and glycosides. LC-MS analysis was carried out to identify the major compounds from each crude extract. A total of 12 phenolic compounds in the extracts of the 3 species were identified and quantified, including 9 flavonoids, 2 hydroxybenzoic acids, and 3 hydroxycinnamic acids. The current study also revealed a good correlation between total phenolic contents and the observed antioxidant activity of the crude extracts.

## 1. Introduction

Medicinal plant species, which have recently grown in popularity, are abundant in different places around the world. They have a high content of bioactive compounds, such as flavonoids, phenolics, anthocyanins, phenolic acids, and non-nutritive and nutritive compounds such as essential oils, vitamins, minerals, etc. Recent research has shown that crude plant species have excellent medicinal value and healthcare functions, something which is also apparent from their familiar usage since ancient times for therapeutic, religious, cosmetic, nutritional, and beautification purposes [1,2,3,4].

Apocynaceae, Asclepiadoideae includes approximately 3000 species in 172 genera and has a worldwide distribution [5]. Many of these species produce cardiac glycosides which have been used for folk medicine and many traditional medical treatments, such as for mental illness and cancer [6,7]. Several species of the family are known as sources of bioactive substances [8] such as alkaloids, terpenoids, and iridoids [9].

Although the physiological process of oxidative metabolism is considered to play a significant role in cell survival, it has noted side effects due to the formation of free radicals and reactive oxygen species (ROS) that can cause damage to DNA, RNA, and protein. However, the human body has a protective mechanism in the form of antioxidants which interact with these products [10].

By definition, antioxidants are chemicals which are used to control oxidative reactions and decrease the adverse effects of reactive species in the biological system due to the low level of natural antioxidants in the body. Therefore, the current research focuses on the potential role of antioxidants and their enzymes in the treatment and prevention of atherosclerosis, heart failure, neurodegenerative disorders, aging, cancer, diabetes mellitus, and several other diseases and/or conditions [10]. Many medicinal plants are known to possess large amounts of polyphenols and potent antioxidant capacity or free radical scavenging activity [11,12,13].

*Calotropis procera* L. is colloquially known as Ishar in certain regions. It grows wild in the Dead Sea and Wadi Araba regions [14]. Additionally, different parts such as root, stem, leaves, flowers, and seeds of *C. procera* are traditionally used to cure several diseases and tackle various symptoms, such as fever, rheumatism, indigestion, cough, cold, eczema, asthma, elephantiasis, nausea, vomiting, leprosy, and diarrhea [15,16]. Phytochemical investigation of this plant has revealed the presence of triterpenes, triterpenoids, phytosterols, saponins, alkaloids and cardiac glycosides [17,18,19,20] in it, and it has also been found to have antioxidant, antimicrobial, and cytostatic properties [21,22,23].

*Pentatropis spiralis* (Forsk.) Decne is distributed in the tropical regions of Asia, Africa, and Australia. In Jordan, it is located in Ghor Al Mazraa [14]. The species contains biological active ingredient compounds such as triterpenes [24,25], which have been used as a remedy for gonorrhea and as a purgative [26,27].

Other poisonous species of the crude plant known as *Peruglaria tomentosa L* are distributed in Saharan and sub-Saharan countries of North Africa [27], including Algeria, Niger, and Egypt [28], and are also common in the Middle East region, including Saudi Arabia and Jordan [14]. This plant has been used extensively in traditional medicine as a depilatory, laxative, and anthelmintic and for skin diseases [29]. Previous phytochemical investigations have focused on checking, isolating, and characterizing several cardenolides, glycosides, flavonoid glycosides, and alkaloids, and β-sitosteryl glucoside [28,29,30,31,32,33,34].

**Figure 1:**
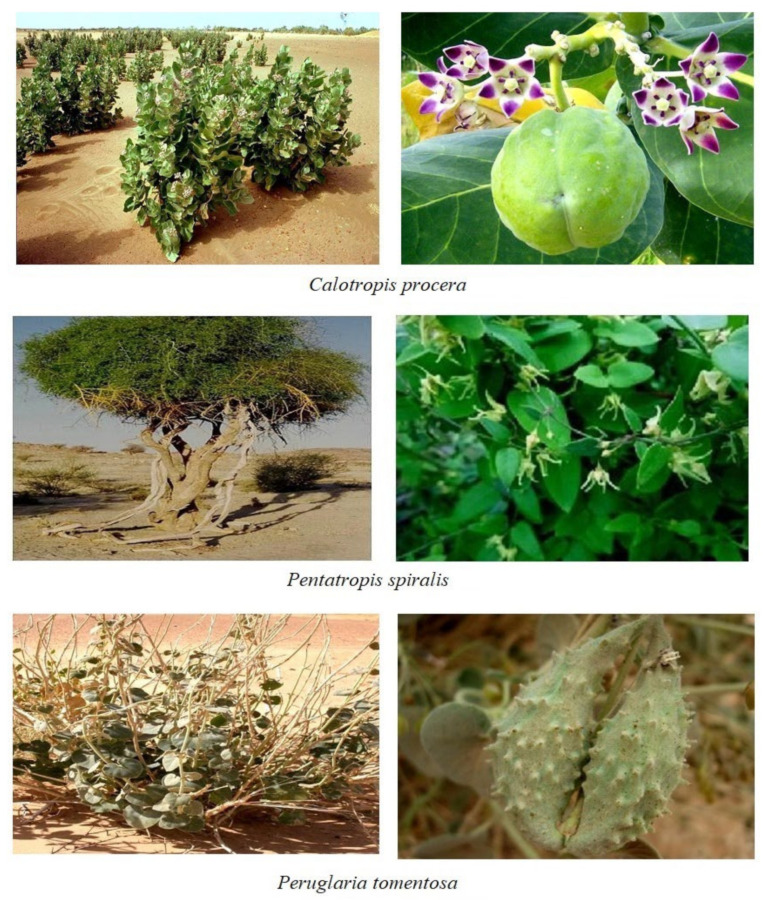
Plants used in this study.

Little information is available regarding the phytochemical constituents and antioxidant activity of *C. procera*, *P. spiralis*, and *P. tomentosa* (Figure 1). Therefore, the present study aimed to evaluate the total phenolic content (TPC) and total flavonoid content (TFC) of butanol, water, and aq. methanol extracts for the three species and then to identify these components using the LC-MS technique. In addition, the study evaluates the antioxidant activity of the three species.

## 2. Results and Discussion

### 2.1. Qualitative Phytochemical Analysis

Qualitative chemical analysis was carried out to ensure the presence of major phytochemical constituents in accordance with the procedures described in the Materials and Methods section of this paper [35]. The results associated with the crude extracts of *C. procera* and *P. Spiralis* (Table 1) indicated that the water fraction was present in most of the tested groups except glycosides and anthraquinones. Alkaloids and terpenes were also observed in all fractions of polar solvent, while flavonoids were absent in the aq. methanol fraction. This pattern is similar to that found in the water fraction of *C. procera.* Except for saponins, where the butanol extract was free of all tested groups. Phytochemical screening tests on crude extract fractions of *P. tomentosa* (Table 1) found that the constituents of the various fractions of *P. tomentosa* were different in their pattern compared with *C. procera* and *P. spiralis.* For instance, *P. tomentosa* was the only plant that contained glycosides, in the form of a water fraction. Moreover, alkaloids were only observed in the aq. methanol fraction of *P. tomentosa*.

### 2.2. LC-MS/MS Analysis of Phytochemicals

LC-MS/MS is a confirmed hyphenated and accurate tool for rapid analysis, and it was used for the identification of a total of 12 phenolic compounds in the *C. procera*, *P. spiralis*, and *P. tomentosa* extracts. In all, 9 flavonoids, 2 hydroxybenzoic acids, and 3 hydroxycinnamic acids were identified by comparing their retention times and mass fragmentation pattern with data obtained from commercial standards, and then each individual compound was quantified by comparing its peak area with the calibration curve obtained for the corresponding standard (Table 2).

Kaempferol-3-*O*-glucoside appeared as the major phenolic subclass found in the butanol extract of *C. procera* (20.36%), whereas 15.83% of *p*-coumaric acid was found in the aq. methanol extract of the same plant. Quercetin, kaempferide, ferulic acid, and 3-glu-7-Rha Quercetin appeared as the predominant compounds in the *P. spiralis* extracts. Kaempferol-3-*O*-glucoside, kaempferol-7-*O*-glucoside, luteolin 7-*O*-glucoside, and 3-hydroxy-4-methoxycinnamic acid were the major compounds in the *P. tomentosa* extracts. The final findings of this study showed that the profiles and contents of phenolic compounds vary depending on the crude extract of the plant.

### 2.3. Total Phenolic Content (TPC) and Total Flavonoid Content (TFC)

The results of TPC and TFC of *C. procera*, *P. spiralis*, and *P. tomentosa* extracts are listed in Table 3. They show concentrated phenols in the polar fractions of the extracts, and the highest levels of phenols and flavonoids were observed in the butanol fraction, followed by the aq. methanol fraction. About 50% of the overall phenols extracted by all solvents was contained in the butanol fraction. Flavonoids showed an almost similar behavior to that of phenols, and most of them were predominant in the butanol extract, followed by the aq. methanol and the water extracts. Uncharacteristically, the content of flavonoids in the butanol extract was extremely high, representing about 86% of the overall flavonoids extracted from all solvents.

### 2.4. Antioxidant Activity

**Figure 2:**
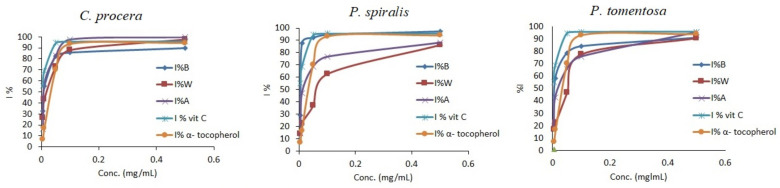
Antioxidant activity of crude extracts from *C. procera*, *P. spiralis*, and *P. tomentosa* and standards using the DPPH method. B = butanol extract, A = aq. MeOH extract, W = water extract.

**Figure 3:**
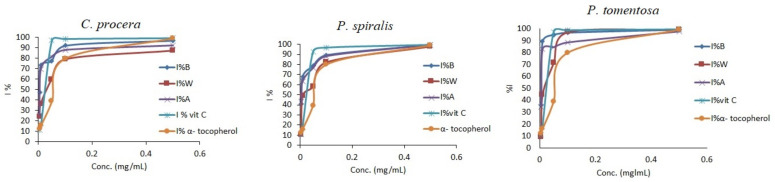
Antioxidant activity of crude extracts from *C. procera*, *P. spiralis*, and *P. tomentosa* and standards using the ABTS method. B = butanol extract, A = aq. MeOH extract, W = water extract.

Various methods have been used to evaluate the free radical scavenging activity of plant extracts [36,37,38,39,40,41,42,43,44,45,46,47,48]. In the present study, four methods were used to evaluate antioxidant activity for the aq. methanol, butanol, and water extracts of three plants: DPPH, ABTS radical scavenging, hydrogen peroxide scavenging (HDR), and ferrous ion chelating activity (FIC) assays (Table 4). The results obtained using the DPPH and ATBS methods clearly indicate a dose-dependent antioxidant activity of the crude extract (Figure 2 and Figure 3).

Furthermore, in Table 4, the results indicate the order of radical scavenging power—for instance, butanol > aq. methanol > water in three species. The DPPH and ABTS scavenging activities of the butanol extracts from *C. procera* (IC_50_ = 0.26 ± 0.02 mg/mL and 0.10 ± 0.01 mg/mL, respectively), *P. spiralis* (IC_50_ = 0.15 ± 0.02 mg/mL and 8.60 × 10^−5^ ± 1.0 × 10^−5^ mg/mL, respectively), and *P. tomentosa* (IC_50_ = 0.35 ± 0.04 mg/mL and 0.11 ± 0.01 mg/mL, respectively) are also shown. These values demonstrate the strong antioxidant activity of the butanol extracts compared with those of *α-tocopherol* and *Vit. C* (Table 4), which may be attributed to butanol extracts’ high TPC and TFC.

**Figure 4:**
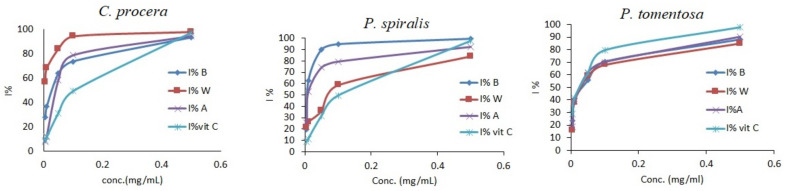
Antioxidant activity of crude extracts from *C. procera*, *P. spiralis*, and *P. tomentosa* and standards using the HDR method. B = butanol extract, A = aq. MeOH extract, W = water extract.

**Figure 5:**
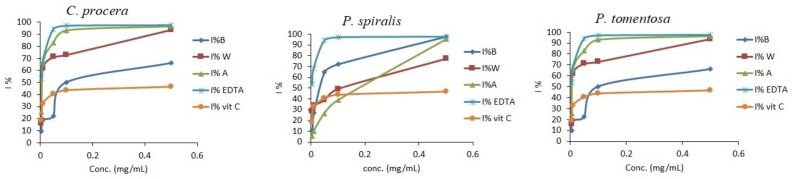
Antioxidant activity of crude extracts from *C. procera*, *P. spiralis*, and *P. tomentosa* and standards using the FIC method. B = butanol extract, A = aq. MeOH extract, W = water extract.

Because of the highly reactive species of hydroxyl radicals toward proteins, lipids, and DNA, with their markedly harmful effects on cell survival when overproduced [49], removal of these types of radicals is particularly important for living systems in order to maintain redox homeostasis. Figure 4 shows the scavenging activities of extracts obtained from *C. procera*, *P. spiralis*, and *P. tomentosa*. It reveals that HDR activity was also concentration-dependent, and again, the W fraction obtained from *C. procera*, B extract obtained from *P. spiralis*, and A extract obtained from *P. tomentosa* were the most active fractions (Figure 4, Table 4). The chelating activities of all crude extracts of *C. procera*, *P. spiralis*, and *P. tomentosa* were also investigated using the FIC method (Figure 5), and the results showed that increases took place in a dose-dependent manner. Based on the IC_50_ values, the water fractions in the three plants had the highest chelating activity (Table 4).

## 3. Materials and Methods

### 3.1. Plant Materials

Fresh aerial parts of *C. procera*, *P. spiralis*, and *P. tomentosa* were collected from Jordan Valley and Aqaba in June 2014. The plants were identified by Jamil N. Lahham, Department of Biology, Faculty of Science, Yarmouk University (*C. procera* (Aiton) Aiton fil (YU/01/AC/1001), P. *spiralis* (Forskal) Decne (YU/09/AP/1001), and *P. tomentosa* L. (YU/10/AP/1001)). All plants were air-dried in shade.

### 3.2. Materials and Equipment

UV spectra were measured using a Biochrom WPA Wave light II UV–visible spectrophotometer. All chemicals used in this investigation were purchased from Sigma-Aldrich (Buchs, Switzerland), including DPPH (2,2-diphenyl-1-picrylhydrazyl), ABTS (2,2′-azino-bis(3-ethylbenzoline-6-sulfonic acid) diammonium salt), Folin & Ciocalteu’s phenol reagent, ferrozin(3-(2-pyridyl)-5,6-diphenyl-1,2,4-triazine-*p,p’*-disulfonic acid monosodium salt hydrate), FeCl_2_ (VWR, Randor, PA, USA), NaOH, AlCl_3_, Na_2_CO_3_, NaNO_2_, H_2_O_2_, FeSO_4_, and salicylic acid (Sigma-Aldrich, Buchs, Switzerland). K_2_S_2_O_8_ was a product of Fluka (Steinheim, Germany).

### 3.3. Extraction and Partitioning

Air-dried and ground plant materials obtained from all plants were extracted using a Soxhlet extractor with petroleum ether to remove fatty acids. After drying, plant residue was extracted using the same apparatus with methanol. The obtained alcoholic gummy residue was then partitioned between CHCl_3_ and H_2_O (1:1) in accordance with the procedures mentioned in the literature [36,37,38,39,40,41,42,43,44,45,46,47,48]. The dried chloroform residue was then subjected to partitioning between 10% aqueous methanol and hexane. Polar organic compounds were extracted from water using *n-*butanol. Thereafter, all fractions obtained were screened for their phytochemical constituents and assayed for their total phenol, total flavonoids, and in vitro antioxidant activities.

### 3.4. Qualitative Phytochemical Analysis

Crude extracts obtained from *C. procera*, *P. spiralis*, and *P. tomentosa* were tested for the presence of flavonoids, alkaloids, tannins, terpenes, saponins, and glycosides in accordance with the procedures described in the literature [35]. Qualitative results expressed as (+) for the presence and (**−**) for the absence of the indicated phytochemical classes are summarized in Table 1.

### 3.5. LC-MS Analysis of Phytochemicals

Metabolite profiling of the crude extracts was carried out using an A Bruker Daltonik (Bremen, Germany) Impact II ESI-Q-TOF system equipped with a Bruker Daltonik Elute UHPLC system (Bremen, Germany) in both positive (M + H) and negative (M − H) electrospray ionization modes (Table 2). Chromatographic separation was performed on a C18 reversed phase column (100 × 2.1 mm, 1.8 µm, 120 Å) from Bruker Daltonik (Bremen, Germany) at 30 °C, with an autosampler temperature of 8 °C and a total run time of 20 min using water/methanol (90:10%) with 5 mM ammonium formate and 0.1% formic acid as an eluent. Plant samples were dissolved with 2.0 mL DMSO; the volume was completed to 50 mL using acetonitrile, then each sample was centrifuged at 4000 rpm for 2 min, and 3.0 µL was injected. The composition of the samples was determined based on the identification of the m/z ratio with a reference to the retention time of the used standards.

### 3.6. Total Phenolic (TPC) and Total Flavonoid Contents (TFC)

TPC was determined using the Folin–Ciocalteu method in accordance with the procedures described in the literature [44,46,48], with slight modifications. Briefly, 0.5 mL of each extract was treated with 2.5 mL of Folin–Ciocalteu reagent (2N) (diluted ten-fold) and 2 mL of Na_2_CO_3_ (75 g/L). The mixture was allowed to stand at room temperature for 15 min, and absorbance was then recorded at 765 nm. Methanol was used as a blank solution. TPC in different extracts of both plants was expressed as mg/g gallic acid equivalent. All measurements were performed in triplicate.

TFC in the different extracts obtained from *C. procera*, *P. spiralis*, and *P. tomentosa* was determined calorimetrically using the aluminum chloride assay method [47,48]. A 1.0 mL aliquot from the stock solution (1 mg/mL) of each extract, diluted in 4.0 mL distilled water, was introduced into a 10.0 mL volumetric flask, to which 0.30 mL of sodium nitrite solution (5% NaNO_2_, *w/v*) was added. The resulting mixture was allowed to stand for 5 min; then, 0.30 mL of aluminum chloride solution (10% AlCl_3_, *w/v*) was added. The resulting solution was incubated for another 6 min, after which 2.0 mL of 1.0 M NaOH solution was added, and the final volume was adjusted to 10.0 mL with distilled water. After 15 min, absorbance was measured at 510 nm. Methanol was used as a blank solution. TFC was measured and performed in triplicate and expressed as mg quercetin/g of dry extract.

### 3.7. Antioxidant and Radical Scavenging Activity

The antioxidant activity of different extracts of *C. procera*, *P. spiralis*, and *P. tomentosa* was screened using the 2,2-diphenyl-1-picrylhydrazyl (DPPH), 2,2′-azino-bis-3-ethylbenzothiazoline-6-sulphonic acid (ABTS), ferrous ion chelating (FIC), and hydroxyl radical assay (HR) methods in accordance with the procedures mentioned in the literature [46,47,48]. Alpha-tocopherol, EDTA, and ascorbic acid were used as positive controls. The IC_50_ of the extracts and positive controls, expressed as mean ± SD, is shown in Table 4. All determinations of the IC_50_ using the three assay methods were conducted in triplicate.

### 3.8. Statistical Analysis

All experiments were conducted independently at least three times. Statistical analysis was performed using PLS Toolbox 4.0 (Eigenvector Research, Inc., Manson, WA, USA) under MATLAB 7.0.4. (MathWorks, Newton, MA, USA). *P* values were determined through one-way ANOVA followed by LSD, and differences were considered significant if *p* < 0.05. Data were expressed as means ± SEM. All data were reported as mean ± SD values.

## 4. Conclusions

This study reports the results obtained from phytochemical analysis performed via LC-MS on crude extracts retrieved from three *Asclepiadaceae* species, namely, *C. procera, P. tomentosa*, and *P. spiralis*. Several flavonoids and phenolic compounds were identified in the extracts, explaining the well-known biological activities of these species. The present study confirmed that the aerial parts of these species from Jordan contain a large amount of kaempferol-3-*O*-glucoside, *p*-coumaric acid, and luteolin-7-*O*-glucoside. In addition, the study revealed that the antioxidant activity of the butanol and aq. methanol extracts of the three species correlates with the highest amount of phenolic and flavonoid contents, which exhibited the greatest antioxidant activity in the scavenging of DPPH free radical, ABTS radical cation, ferrous chelating effect, and hydroxyl radical assays. These results indicate that the butanol extracts of these species may be useful as biologically active agents in food and pharmaceutical formulations since they are rich in phenolic and flavonoid compounds. 

## Figures and Tables

**Table 1:** Major phytochemical groups detected in crude extract fractions of *C. procera*, *P. spiralis* and *P. tomentosa*.

Groups	*C. procera*			*P. spiralis*			*P. tomentosa*		
	B	A	W	B	A	W	B	A	W
Alkaloids	+	+	+	-	+	+	-	+	-
Tannins	+	-	+	-	+	+	+	-	+
Flavonoids	+	-	+	-	-	+	+	+	-
Saponins	-	+	+	+	+	+	-	+	-
Anthraquinone	-	-	-	-	-	-	-	-	-
Glycosides	-	-	-	-	-	-	-	-	+
Terpenoids				-	+	+	+	-	-

B = butanol extract, A = aq. MeOH extract, W = water extract.

**Table 2:** LC-QTOF-MS/MS analysis data of detected metabolites from the crude extracts of *C. procera*, *P. spiralis*, and *P. tomentosa* extracts.

No.	R_t_ Min	Compound	Relative Percentage Amounts (%)								
			*C. procera*			*P. spiralis*			*P. tomentosa*		
			B	A	W	B	A	W	B	A	W
1	2.57	4-Hydroxybenzoic acid	2.31	11.23	-	-	-	-	3.67	13.62	-
2	3.58	Syringic acid	-	5.36	-	-	-	0.29	-	-	-
3	4.45	*p*-Coumaric acid	5.21	15.83	0.14	0.82	2.38	-	4.26	-	0.29
4	5.15	Ferulic acid	6.42	-	0.78	10.20	-	-	2.31	-	-
5	5.16	3-Glu-7-Rha Quercetin	2.24	-	8.64	7.36	3.12	10.88	-	-	-
6	5.44	3-Hydroxy-4-methoxycinnamic acid	-	-	5.88	-	-	-	3.61	6.13	10.88
7	5.77	Spiraeoside	1.45	9.01	-	3.92	6.41	-	-	-	-
8	6.26	Luteolin 7-*O*-glucoside	6.51	1.45	-	-	-	4.13	11.91	2.45	-
9	6.54	Kaempferol-3-*O*-glucoside	20.36	5.12	4.13	1.11	2.59	-	21.63	-	4.13
10	6.99	Kaempferol-7-*O*-glucoside	8.41	-	0.29	-	-	-	16.90	-	0
11	8.53	Quercetin	-	3.85	-	0.60	9.60	-	-	4.76	-
12	10.08	Kaempferol	-	-	-	0.31	24.79	-	-	-	-
13	10.47	Isorhamnetin	9.14	-	-	2.64	7.77	-	-	-	-
14	13.89	Kaempferide	-	-	-	8.46	7.56	-	-	-	-

B = butanol extract, A = aq. MeOH extract, W = water extract.

**Table 3:** TPC and TFC of all extracts of *C. procera*, *P. spiralis* and *P. tomentosa*.

Extracts	TPC (mg/g Gallic Acid)			TFC (mg/g Quercetin)		
	*C. procera*	*P. spiralis*	*P. tomentosa*	*C. procera*	*P. spiralis*	*P. tomentosa*
B	377.2 ± 2.6	113.2 ± 2.3	311.50 ± 3.04	82.7 ± 1.3	168.5 ± 0.9	107.7 ± 1.5
A	126.4 ± 4.5	59.2 ± 1.9	213.00 ± 1.32	52.3 ± 1.8	112.1 ± 1.1	69.9 ± 1.6
W	181.5 ± 2.5	30.8 ± 1.6	62.00 ± 1.80	28.2 ± 2.8	104.3 ± 0.8	21.0 ± 0.8

Values expressed are means ± S.D. of three parallel measurements: B = butanol extract, A = aq. MeOH extract, W = water extract.

**Table 4:** IC_50_ (mg/mL) values of crude extracts from *C. procera*, *P. spiralis*, and *P. tomentosa* and standards using the DPPH, ABTS, HDR, and FIC methods.

Plant	Crude	DPPH	ABTS	HDR	FIC
*C. procera*	Butanol	0.26 ± 0.02	0.10 ± 0.01	0.52 ± 0.01	1.31 ± 0.07
	Aq. methanol	0.35 ± 0.01	0.25 ± 3.0 × 10^−3^	0.86 ±0.02	0.85 ± 0.03
	Water	0.40 ± 0.01	0.58 ± 0.02	0.06± 0.01	0.09 ± 0.01
*P. spiralis*	Butanol	0.15 ± 0.02	8.60 × 10^−5^ ± 1.0 × 10^−5^	0.29 ± 0.03	0.72 ± 0.02
	Aq. methanol	0.54 ± 0.04	0.07 ± 3.0 × 10^−3^	0.37 ± 0.02	1.77 ± 0.13
	Water	1.16 ± 0.01	0.48 ± 0.01	1.16 ± 0.03	1.72 ± 0.02
*P. tomentosa*	Butanol	0.35 ± 0.04	0.11 ± 0.01	0.88 ± 0.02	2.67 ±0.23
	Aq. methanol	0.54 ± 0.01	0.15 ± 0.01	0.43 ± 0.09	0.26 ± 0.03
	Water	0.83 ± 0.04	0.49 ± 0.02	0.72 ± 0.04	0.44 ± 0.01
Ascorbic acid (Vit. C)		1.78 × 10^−3^ ± 6.0 × 10^−5^	1.58 × 10^−3^ ± 3.0 × 10^−5^	2.6 × 10^−3^ ± 3.0 × 10^−5^	1.89 × 10^−3^ ± 2.00 × 10^−5^
α-tocopherol		2.33 × 10^−3^ ± 4.0 × 10^−5^	1.79 × 10^−3^ ± 1.0 × 10^−5^	-	-
EDTA		-	-	0.013 ± 1.5 × 10^−5^	0.02 ± 1.1 × 10^−5^

## Data Availability

Not applicable.

## Sample Availability

Samples of the compounds are not available from the authors.
